# The mTORC2 Regulator Homer1 Modulates Protein Levels and Sub-Cellular Localization of the CaSR in Osteoblast-Lineage Cells

**DOI:** 10.3390/ijms22126509

**Published:** 2021-06-17

**Authors:** Mark S. Rybchyn, Tara Clare Brennan-Speranza, David Mor, Zhiqiang Cheng, Wenhan Chang, Arthur D. Conigrave, Rebecca S. Mason

**Affiliations:** 1 Discipline of Physiology, School of Medical Sciences and Bosch Institute, Faculty of Medicine and Health, University of Sydney, Sydney, NSW 2006, Australia; m.rybchyn@unsw.edu.au (M.S.R.); tara.speranza@sydney.edu.au (T.C.B.-S.); 2 School of Chemical Engineering, University of New South Wales, Sydney, NSW 2033, Australia; 3 School of Public Health, University of Sydney, Sydney, NSW 2006, Australia; 4 Discipline of Anatomy and Histology, School of Medical Sciences, Faculty of Medicine and Health, University of Sydney, Sydney, NSW 2006, Australia; david.mor@sydney.edu.au; 5 School of Medicine, University of California, San Francisco, CA 94121, USA; czq6527@gmail.com (Z.C.); Wenhan.Chang@ucsf.edu (W.C.); 6 School of Life and Environmental Science, Charles Perkins Centre (D17), University of Sydney, Sydney, NSW 2006, Australia; arthur.conigrave@sydney.edu.au

**Keywords:** CaSR, Homer1, mTOR, osteoblast, osteocyte, AKT, chaperone function

## Abstract

We recently found that, in human osteoblasts, Homer1 complexes to Calcium-sensing receptor (CaSR) and mediates AKT initiation via mechanistic target of rapamycin complex (mTOR) complex 2 (mTORC2) leading to beneficial effects in osteoblasts including β-catenin stabilization and mTOR complex 1 (mTORC1) activation. Herein we further investigated the relationship between Homer1 and CaSR and demonstrate a link between the protein levels of CaSR and Homer1 in human osteoblasts in primary culture. Thus, when siRNA was used to suppress the CaSR, we observed upregulated Homer1 levels, and when siRNA was used to suppress Homer1 we observed downregulated CaSR protein levels using immunofluorescence staining of cultured osteoblasts as well as Western blot analyses of cell protein extracts. This finding was confirmed in vivo as the bone cells from osteoblast specific *CaSR*−/− mice showed increased Homer1 expression compared to wild-type (wt). CaSR and Homer1 protein were both expressed in osteocytes embedded in the long bones of wt mice, and immunofluorescent studies of these cells revealed that Homer1 protein sub-cellular localization was markedly altered in the osteocytes of *CaSR*−/− mice compared to wt. The study identifies additional roles for Homer1 in the control of the protein level and subcellular localization of CaSR in cells of the osteoblast lineage, in addition to its established role of mTORC2 activation downstream of the receptor.

## 1. Introduction

In bone, the roles of canonical Wnts (e.g., Wnt3a) in activating Low-density lipoprotein receptor-related protein 5/6 (LRP5/6) and Frizzled-dependent osteoblastogenesis via β-catenin activation and transfer to the cell nucleus is well established [1]. The roles of several key regulators have been identified, including the osteocyte-derived inhibitor sclerostin, whose levels fall in response to mechanical stimuli [2]. However, the existence and identity of nutrient-dependent signaling pathways that lead to β-catenin stabilization and contribute to this signaling pathway, are largely unknown.

We previously demonstrated that activation of the nutrient-sensing class C G-protein-coupled receptor (GPCR), the Calcium-sensing receptor (CaSR), by Sr^2+^ in human osteoblasts promotes a novel Akt-dependent signaling pathway upstream of Wnt and its canonical mediator of osteoblastogenesis, β-catenin [3]. More recently, while investigating the mechanism that supports this pathway, we demonstrated that the CaSR also mediates osteoblastogenesis in response to the key physiologically regulated divalent cation Ca^2+^ and uncovered roles for Homer1 and mechanistic target of rapamycin complex (mTOR) complex 2 (mTORC2) downstream of the Ca^2+^-stimulated CaSR and upstream of Akt [4].

Several lines of evidence identify CaSR as a key determinant of cell fate and function in bone. Thus, the CaSR is expressed and functional in key bone cell lineages including: pre-osteoblasts/osteoblasts, in which it promotes maturation, proliferation and bone formation; and osteoclasts, in which it suppresses bone resorption [3,5,6,7,8,9,10]. Furthermore, in mice, type-I collagen promoter-directed tissue-specific deletion of CaSR exon-7, which encodes the receptor’s heptahelical domain and intracellular C-terminus, resulted in a severe disturbance of skeletal development and bone mass featuring growth retardation, undermineralization and pathological fractures [5].

The CaSR is a class C GPCR that is closely related to the metabotropic glutamate receptors (mGluRs). Homer proteins act as scaffolds for the mGluR proteins, promoting the assembly of signaling complexes permitting the activation of growth and survival pathways such as AKT and ERK [11,12,13]. We have recently shown that Homer also directly interacts with CaSR and mTORC2 in osteoblasts, resulting in AKT phosphorylation [4]. Given the direct interaction of Homer and CaSR proteins, we have further predicted that Homer proteins may exert a chaperone-like function on CaSR, thereby promoting stability of CaSR in the cell.

In the current study, we set out to determine whether Homer1 stabilizes and promotes CaSR protein levels in bone cells. We have assessed whether CaSR and Homer1 might impact each other’s expression at the protein level using siRNA to suppress separately CaSR and Homer1 in human osteoblasts in primary culture, compared Homer1 expression levels in conditional osteoblast-specific *CaSR*-null mice, and extended the analyses to include osteocytes as well as osteoblasts.

## 2. Results

### 2.1. CaSR and Homer1 Were Required for Extracellular Ca^2+^-Dependent AKT and GSK3β Phosphorylation in Immortalized Primary Human Osteoblasts and Promoted Osteoblast Viability

**Figure 1:**
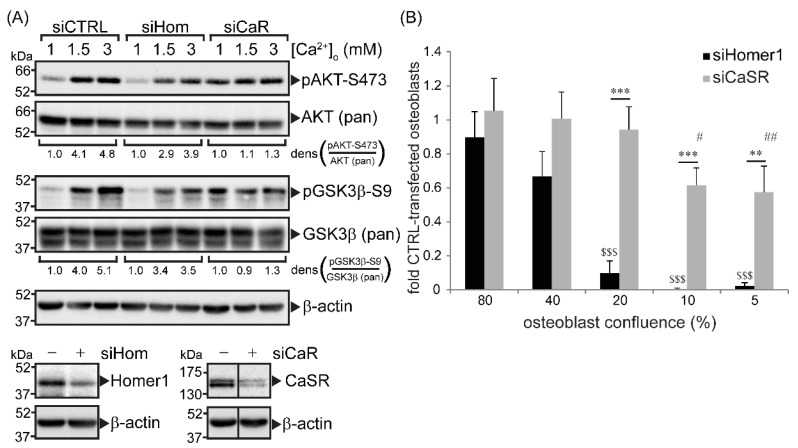
Calcium-sensing receptor (CaSR) and Homer1 modulated extracellular Ca^2+^-dependent phosphorylation of AKT and glycogen synthase kinase-3 (GSK3) and cell viability in human osteoblasts. (**A**), top: Western blot showing the level of phosphorylated AKT (S473), total AKT, phosphorylated GSK3 (S9), total GSK3 and β-actin (loading control) in response to extracellular Ca^2+^ concentrations for 15 min in simian virus 40 (SV40)-immortalized primary human osteoblasts. Prior to Ca^2+^ treatment, cells were transfected with siRNA targeted at Homer1 (siHom), CaSR (siCaR) or a non-directed sequence (siCTRL) for 48 h. Densitometric values (dens) were calculated (Image J) by expressing the value obtained for phosphorylated protein by the total protein for each treatment and normalizing this number to the ratio obtained from the 1 mM Ca^2+^ lane for each silenced condition (vehicle treatment). (**A**), bottom: Western blot showing the protein level of Homer1 and CaSR in response to each targeted siRNA (+) compared with cells transfected with non-directed sequence (−) in immortalized primary human osteoblasts. Solid, vertical black lines on the presented Western blotting (CaSR blot only) indicate where lanes have been spliced from the same Western blotting for presentation purposes. (**B**) Primary human osteoblasts were plated to achieve the indicated degree of confluence after 24 h. Cells were subsequently transfected with siRNAs directed against Homer1, CaSR or a non-directed sequence. Following a further 48 h culture, cell viability was quantified using the resazurin dye CellTiterBlue™. The data are presented as fold viabilities with respect to the viability of osteoblasts transfected with the non-directed sequence. $$$ *p* < 0.001 compared to cells at 80% confluence transfected with siHomer1; # *p* < 0.05, ## *p* < 0.01 compared to cells at 80% confluence transfected with siCaSR; ** *p* < 0.01, *** *p* < 0.001 compared as indicated with horizontal line.

The effect of Homer1 and CaSR on AKT-Ser^473^ and glycogen synthase kinase-3β (GSK3β)-Ser^9^ phosphorylation in response to extracellular Ca^2+^ was measured in immortalized primary human osteoblasts. siRNA-directed knockdown of either Homer1 or CaSR protein (Figure 1A) resulted in the suppression of Ca^2+^-induced phosphorylation of AKT and GSK3β, compared to control cells transfected with a non-directed sequence (Figure 1A). This finding was in agreement with our previous studies carried out in non-transformed primary human osteoblasts [3,4] and MG63 osteosarcoma cells [4].

We predicted that, as AKT is a critical control point for cell growth and survival, silencing Homer1 and CaSR would result in significant reductions in osteoblast viability. Primary human osteoblasts were seeded at several densities and permitted to attach for 24 h. Lowering cell densities is an established method to induce stress in primary cells due to several factors, including reductions in autocrine growth factors [14]. Cells were subsequently transfected with siRNA directed at Homer1, CaSR or a non-directed sequence (control). At an established time point of siRNA targeted protein reduction (shown in Figure 1A), the resazurin dye Cell Titer Blue™ was added as a measure of cell viability. siRNA directed at Homer1 and CaSR reduced osteoblast viability (Figure 1B), however, silencing Homer1 yielded greater reductions in viability compared to CaSR at all cell densities tested and resulted in no viable osteoblasts where plating densities of 10% or less were used (Figure 1B).

### 2.2. Protein Levels of Homer1 and CaSR Were Linked in Primary Human Osteoblasts

**Figure 2:**
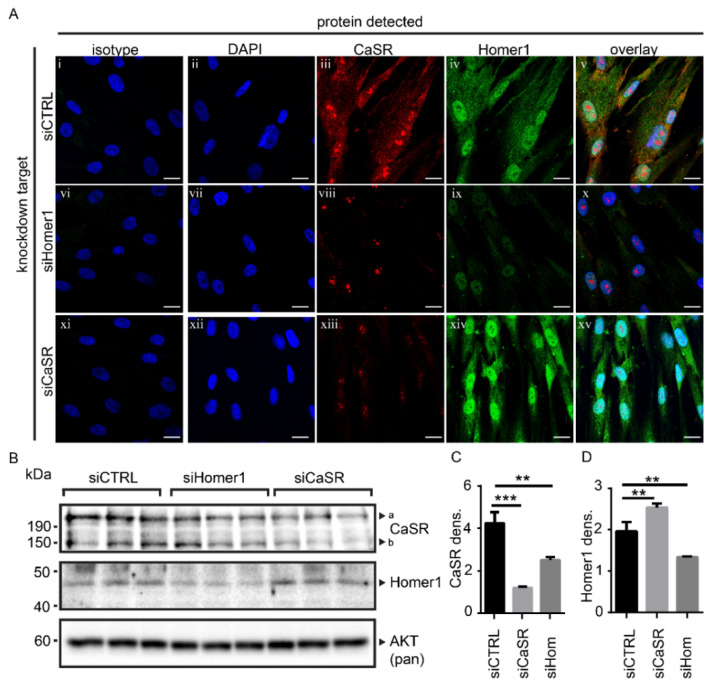
The protein levels of Homer1 and CaSR were linked in osteoblasts in vitro (**A**) (**i**–**xv**) Confocal microscopy of osteoblasts transfected with a non-directed control siRNA (siCTRL, (**i**–**v**)) or siRNA to Homer1 (siHomer1, (**vi**–**x**)) or CaSR (siCaSR, (**xi**–**xv**)). Cells stained for Homer1 (green), CaSR (red) in combination with nuclear counterstain 4′,6-diamidino-2-phenylindole (DAPI) (blue). The combined channels of CaSR, Homer1 and DAPI is shown in **v**,**x**,**xv** for each silenced condition (overlay). The combined isotype control stain is shown in **i**, **vi** and **xi** for each silenced condition (isotype). 63× objective, Scale bar = 10 μm. (**B**) Western blot of CaSR, Homer1 or total AKT (loading control) from total osteoblast lysates of cells 48 h post transfection with siRNA to Homer1 (siHomer1), CaSR (siCaSR) or control sequence (siCTRL)–each from triplicate wells (individual wells run in individual lanes); “a” the higher molecular weight CaSR band is most likely heterodimerized CaSR [20]; “b” is the normal glycosylated molecular mass for CaSR ~150 kDa. (**C**,**D**) Densitometry of triplicate bands shown in “B” for (**C**) CaSR, or (**D**) Homer1 protein levels. ** *p* < 0.01, *** *p* < 0.001.

Homer1 and CaSR proteins were detected in cultured primary human osteoblasts by immunofluorescence (Figure 2A(ii–v)). Since Homer1 modulates the expression of several other known binding proteins [15,16], we tested whether reducing Homer1 protein affected CaSR protein levels. Confocal microscopy showed that knockdown of Homer1 by siRNA reduced Homer1 (Figure 2A(iv, ix)), and also reduced CaSR protein levels compared to control transfected cells (Figure 2A(iii,viii)). Conversely, siRNA-mediated knockdown of the CaSR, whilst reducing CaSR protein levels as expected (Figure 2A(iii,xiii)), *increased* protein levels of Homer1 (Figure 2A(iv,xiv)). While the localization of Homer1 and CaSR by immunofluorescence appear to be nuclear due to the strong overlap with the 4′,6-diamidino-2-phenylindole (DAPI) counterstain, this is highly likely to be endoplasmic reticulum (ER)-resident protein. The ER is a native intracellular pooling site for CaSR [17,18] where it has been shown to dimerize [19].

Western blot analysis was used to confirm the relationship between Homer1 and CaSR proteins shown by immunofluorescence in Figure 2A,B. Western blot was in agreement with the immunofluorescence data, where siRNA directed at Homer1 decreased Homer1 protein (*p* < 0.01) and CaSR protein (*p* < 0.01) compared to control transfected cells. siRNA directed at CaSR significantly reduced CaSR protein (*p* < 0.001) but resulted in *higher* Homer1 protein levels (*p* < 0.01) (Figure 2B). Densitometry for Western blot data was performed (Image J) to generate the statistical values listed above and is shown in Figure 2C,D.

### 2.3. Homer1 Protein Expression Was Increased in the Long Bones of Osteoblast-Specific CaSR Knockout Mice

**Figure 3:**
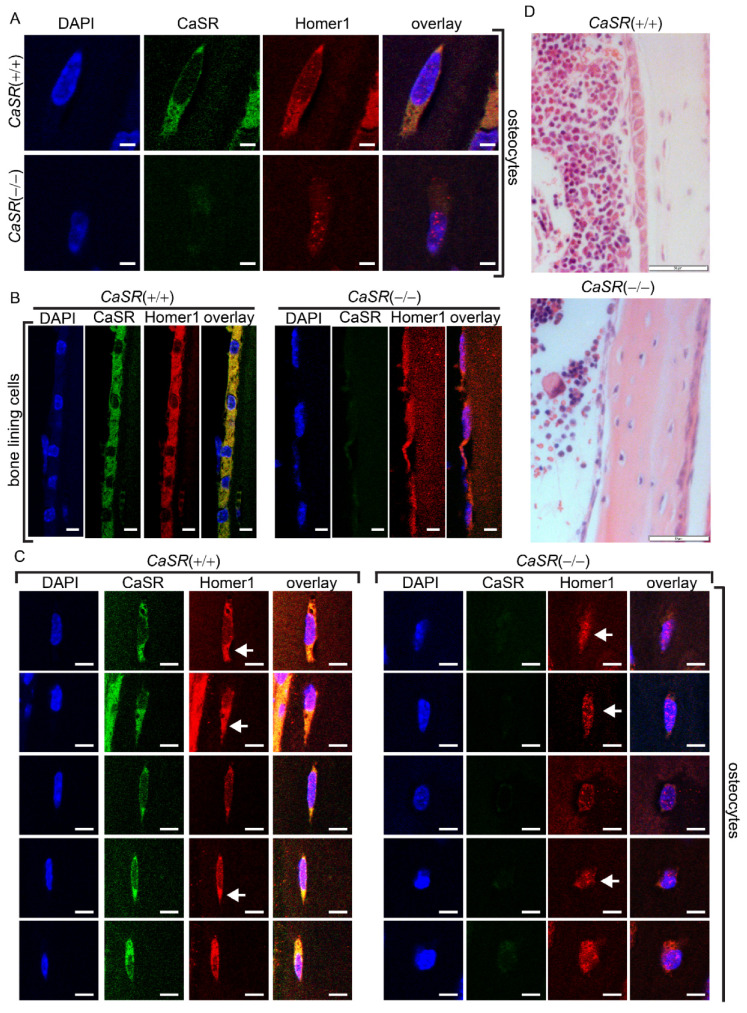
The protein levels of Homer1 and CaSR were linked in bone cells in vivo, and the sub-cellular distribution of Homer1 was modulated by CaSR. (**A**,**B**) Confocal microscopy of (**A**) an osteocyte embedded in the mineralized matrix or (**B**) bone lining cells of long bones of wt (*CaSR*+/+), or CaSR knockout (*CaSR*−/−) mice, stained for Homer1 (red) or CaSR (green). Cell nuclei (blue) stained by DAPI, 100× objective (scale bar = 5 μm). (**C**) Confocal microscopy of several individual osteocytes from the long bones of *CaSR*+/+ and *CaSR*−/− mice stained for Homer1 (red) or CaSR (green). Cell nuclei (blue) stained by DAPI, 100× objective (scale = 5 μm). White arrows indicate examples of changes in Homer1 cellular localization compared to wt between CaSR phenotypes. (**D**) H&E stain of long bones of wt (*CaSR* (+/+)) or CaSR knockout (*CaSR*(−/−)) mice. Scale bar = 50 μm.

**Figure 4:**
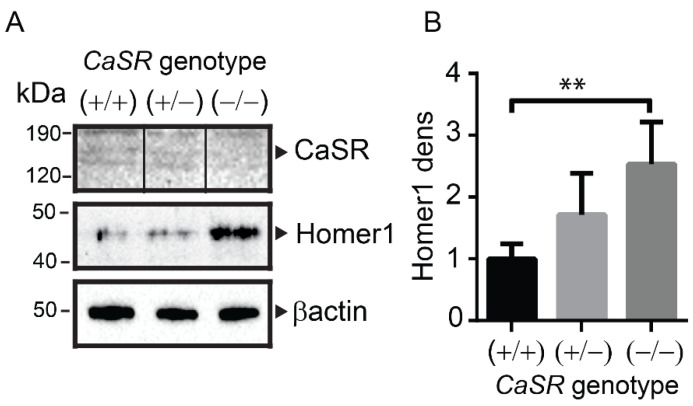
(**A**) Western blot of protein extracted from mouse long bones from wt (*CaSR*+/+), heterozygous (*CaSR*+/−) or CaSR knockout (*CaSR*−/−). β-actin loading control. The faint bands present for CaSR are due to the inefficient extraction of transmembrane associated proteins such as CaSR by non-detergent based chemical extraction of total bone Solid, vertical black lines on the presented Western blotting (**A***,* CaSR blot) indicate where lanes have been spliced from the same Western blotting for presentation purposes. (**B**) Densitometry of triplicate blots shown in “A” for Homer1 shown as fold of the level in wt (*CaSR*+/+). ** *p* < 0.01.

Confocal microscopy was used to detect protein levels of Homer1 and CaSR in osteocytes (Figure 3A) and bone lining cells (Figure 3B) of wt (*CaSR*+/+) or osteoblast specific *CaSR* null (*CaSR*−/−) mice [5]. As expected, no CaSR protein was detected in bone lining cells or osteocytes from *CaSR* null mice. To quantify Homer1 protein levels from the various *CaSR* genotypes, total protein was chemically extracted from the long bones of *CaSR*+/+, *CaSR*+/− and *CaSR*−/− animals, and then subjected to Western blot analysis (Figure 4A). Homer1 was significantly higher in *CaSR*−/− mice compared to *CaSR*+/+ (Figure 4A,B *p* < 0.01). In *CaSR*+/− mice there was a non-significant increase in Homer1 when compared to *CaSR*+/+ (Figure 4). As expected, CaSR, as a membrane protein, was not readily solubilized with guanidine-based chemical extraction of total bone, though the CaSR was clearly detected in wt, and was below the level of detection for the *CaSR*−/− protein extract (Figure 4A) and overall the findings on CaSR protein levels were visually consistent with those of the immunofluorescence analyses (Figure 3). Taken together these in vitro and in vivo findings indicate that the protein levels of CaSR and Homer1 are interdependent.

### 2.4. CaSR Modulated the Sub-Cellular Localization of Homer1 in Osteoblast-Lineage Cells In Vivo

We have previously shown that CaSR and Homer1 proteins co-immunoprecipitate in vitro [4]. The confocal microscope images shown in Figure 3A show that while wt osteocytes exhibited a very similar cytoplasmic distribution of both CaSR and Homer1, the *CaSR*−/− phenotype resulted in Homer1 staining that localized to and around the nucleus, which likely represented accumulation of the protein in the ER where Homer1 has previously been shown to natively reside within the cell [21,22,23,24] (Figure 3C). Multiple images of osteocytes from the long bones of both *CaSR*+/+ and *CaSR*−/− mice, shown in Figure 3C, show the change in Homer1 cellular localization described above was also consistently observed in osteocytes between *CaSR* wt and null phenotypes (Figure 3C, white arrows), and was also observed in bone lining cells (Figure 3B).

## 3. Discussion

Given the importance of the CaSR in the development of bone phenotype and for mediating the positive effects of dietary calcium on bone [5,6,7,25], the observation that Homer1 is required for normal CaSR protein levels and is critical for CaSR function in osteoblasts [4] points to the existence of previously unknown roles for Homer1 in skeletal development and maintenance, and possibly in other tissues as CaSR is ubiquitously expressed.

To our knowledge, this is the first example of an increase in Homer1 arising from decreased levels of one of its binding proteins. The observation that CaSR protein levels depend in part, on Homer1 has also not been previously reported. Although the mechanism by which the normal presence of Homer1 increases CaSR protein levels is unclear, it seems plausible that in the process of forming stable protein complexes, Homer1 stabilizes the total cellular pool of CaSR protein and slows the rate at which it is directed to lysosomes for degradation [26]. Previously, over-expression of Homer1b was reported to perturb mGluR5 protein trafficking and/or expression with different effects in different cell types [27,28].

The findings in the present study suggest that the protein levels of CaSR and Homer1 are co-regulated. From the available data we are unable to determine whether the co-modulation of Homer1 and CaSR is due to protein stability or transcript expression; however, a clear relationship exists at the protein level which is the critical point for assessing a role for the two proteins in cellular function. Within this study, we did also look at mRNA levels in primary human osteoblasts in response to siRNA directed at both Homer1 and CaSR. Unfortunately, we did not observe consistency across osteoblasts donors with these analyses, and therefore we did not present mRNA data in the current study. Nevertheless, we propose that, ultimately, the important finding here is at the protein level, and potential transcription effects, are not essential to the study’s conclusions.

Although this study focused on the long form of Homer1 (commonly termed the canonical sequence “Homer1b” or “Homer1c”; Uniprot identifier Q86YM7-1), we also detected the short form of Homer1, Homer-1a (Q86YM7-3), and other protein expression of the closely related Homer2 (Q9NSB8) and Homer3 (Q9NSC5) in primary human osteoblasts and the protein levels detected were consistent across primary cell donors (Supplementary Figure S1). Given the high degree of homology between these proteins, it is possible that functional crossover, or redundancy of function, does occur. Importantly, this may explain the lack of a reported bone phenotype in Homer1 knockout mice [29,30,31]. In support of this argument, we observed that Homer2 protein levels in bone were also altered by *CaSR* knockout in mice, compared to wt, and importantly, this change in protein level was in the opposite direction to Homer1, suggesting a compensation mechanism may exist in osteoblasts between various Homer isoforms (Supplementary Figure S2). Therefore, it is plausible to suggest that the expression level of other Homer isoforms could be modulated in bones of Homer1 knockout mice to provide compensatory function for the loss of Homer1. Although no bone phenotype has been reported in the mouse model, recently it has been shown that the *Homer1* gene is linked to the positive regulation of bone mass in postmenopausal women [32].

Also shown in this study, silencing Homer1 or CaSR inhibited primary human osteoblast viability and we have previously demonstrated that Homer1 and CaSR promote differentiation and inhibit apoptosis in this cell type [4]. Positive modulation of extracellular Ca^2+^-induced AKT phosphorylation by both CaSR and Homer1 shown herein was in agreement with previous studies which also examined the broader intracellular signaling cascade [3,4]. Taken together with the central finding of this study, that the protein levels of CaSR and Homer1 in osteoblasts are interdependent, it appears reasonable to propose that Homer1 is likely critical to CaSR function in bone via several mechanisms. However, a more detailed investigation of the bones of *Homer1* knockout mice specifically investigating the possibility of redundancy of protein function (such as Homer2 functional redundancy for knockouts lacking Homer1 as proposed above) would be necessary to further establish this idea.

In preliminary analyses we also found that a correlation existed between the native protein levels of CaSR and Homer1 in the commonly used osteosarcoma cell lines MG63 (ATCC^®^ CRL-1427™) and SAOS-2 (ATCC^®^ HTB-85™). We also compared the protein levels in the osteosarcoma lines to that present in primary human osteoblasts (Supplementary Figure S3). Both osteosarcoma cell lines exhibited markedly enhanced Homer1 expression compared to primary osteoblasts, along with increased expression of CaSR, mTOR, the mTORC2 specific protein Rictor, but not the mTORC1 specific Raptor, and the effects were particularly pronounced in SAOS-2. Whether increased CaSR/Homer1 expression and resultant AKT activation contributes to the high survival, high growth phenotype of osteosarcoma cells and are thus potential targets for the chemotherapy of osteosarcoma, remains to be determined. A limitation of the study was that rescue experiments were beyond the scope of this study and mesenchymal stem cells from *CaSR*+/+ or *CaSR*−/− mice were not available to allow comparison of the expressions of CaSR, Homer1, mTOR and Rictor in native cells.

In the current study we utilized a mixture of commercially available siRNA duplexes which effectively reduced targeted gene expression at the protein level, and we also verified reductions at the mRNA level (Supplementary Figure S4). When CaSR protein was reduced in cells via siRNA or in *CaSR*−/− mice, we observed a pronounced increase in levels of Homer1 protein, consistent with the proposal that full, normal CaSR function and expression requires Homer1. We propose that the increased expression of Homer1 in response to reduced CaSR protein is a compensatory mechanism of the cell. Beyond the high degree of co-localization of Homer1 and CaSR in bone cells [4], an analysis of Homer1 protein distribution in *CaSR*−/− osteocytes and bone surface lining cells of mice showed that in addition to general thinning of the bone lining cells, Homer1 sub-cellular localization was clearly altered in a manner consistent with its accumulation in the ER in the absence of the CaSR. Retention of Homer1 in the ER has also been reported in the absence of mGluR1 or 5 [33], and likely represents a shift in Homer1 cellular distribution in response to the absence of a native binding protein.

We previously found that CaSR-dependent AKT stimulation in human osteoblasts promoted β-catenin stabilization and subsequent nuclear translocation that was dependent, in part, on the inhibition of GSK3β activity via Ser^9^ phosphorylation, and that the direct chemical inhibition of AKT halted this process [3]. Furthermore, we recently showed that this AKT-dependent process required Homer1, which links CaSR to mTORC2–the point of AKT initiation [4]. The findings of the present study show that, in addition to a direct effect on mTORC2/AKT signaling, Homer1 also regulates CaSR protein level in bone cells.

In conclusion, here we show that the long form of Homer1 positively regulated CaSR protein levels in primary human osteoblasts. Furthermore, in the absence of CaSR, Homer1 protein levels were increased and its sub-cellular localization was clearly altered. These effects are reported using both in vitro and in vivo models. The findings support the hypothesis that, in addition to roles in supporting neuromodulation and neuroplasticity by type-1 metabotropic glutamate receptors, Homer1 maintains the protein level and AKT/mTOR signaling capacity [4] of another nutrient-sensing GPCR of the same family, CaSR. The study supports the hypothesis that Homer proteins play more general roles outside the CNS in controlling the activity of family C GPCRs.

## 4. Materials and Methods

All chemicals, including culture media were obtained from Sigma-Aldrich (St. Louis, MO, USA) unless otherwise specified.

### 4.1. Cell Culture

The study was conducted using human osteoblasts from several different donors. Each experiment was conducted using human osteoblasts from at least two different donors, and cells were maintained routinely in DMEM containing 10% (*v*/*v*) FCS. Human osteoblasts were grown from the minced trabecular ends of fetal long bone in accordance with the National Health and Medical Research Council guidelines and with the approval of the University of Sydney Human Ethics Committee (approval number: 2012/043), as described previously [34]. These bone-derived cells were moderately differentiated as previously described [3]. Because the bone cells were from different donors, some biological variations in responses were observed, as expected [3,4,34,35,36]. SAOS-2 and MG63 cell lines were maintained under similar conditions. SV40-immortalized osteoblasts were transformed by transfection of primary human osteoblasts (described above) with the SV40 early region genes [37]. The transformed osteoblasts displayed the same phenotypic characteristics previously reported for the non-transformed cell type.

### 4.2. CaSR Knockout Mice

Male and female mice with ablation of both alleles of *CaSR* gene in C57/B6 background were generated by breeding the floxed-*CaSR* mice and the mice expressing Cre recombinase transgene under the control of a 2.3 kb promoter of rat type I collagen α-subunit, as described previously [5]. There were no sex differences reported for *CaSR* knockout or wt mice [5,6]. All mice were housed in a pathogen-free climate-controlled room (22 °C; 50% relative humidity) with a 12/12-h dark/light cycle, given filtered water and standard chow containing 1.0% calcium and 0.7% phosphate, and euthanized at 3 months of age by isoflurane overdose for subsequent harvests of serum and bone samples. All animal procedures were approved by the Institutional Animal Care and Use Committee at the San Francisco Veterans Affairs Medical Center.

Femurs and tibiae from wild type, heterozygous and homozygous osteoblast-specific *CaSR* knockout mice [5] were used for immunofluorescent and Western blot analyses of CaSR and Homer isoforms. Femurs were snap frozen and kept at -80 °C prior to protein extraction for Western blot analysis. Tibiae were cleaned, fixed in 10% formalin, decalcified and sectioned at 8 microns thick for immunofuorescence analysis. Sections were stained with haemotoxylin and eosin or for immunofuorescence as described below.

### 4.3. Immunofluorescence (Mouse Bones)

Tibial sections were deparaffinized, cleared in xylene, rehydrated in ethanol steps and permeabilized in Triton X-100 (0.5%). Bone sections were then blocked in 2% bovine serum albumin for 30 min before being probed with anti-CaSR (goat polyclonal, Santa Cruz Biotechnology clone F-19) or anti-Homer-1b/c (rabbit polyclonal, Santa Cruz Biotechnology clone H-174) followed by anti-goat Alexa Fluor 488 (1:750; Santa Cruz Biotechnology, Dallas, TX, USA) and anti-rabbit-Cy3 (1:750; Life Technologies, Carlsbad, CA, USA). To visualize the nuclei, coverslips were mounted with UltraCruz™ Mounting Medium containing 4′,6-diamidino-2-phenylindole (DAPI) (Santa Cruz Biotechnology).

### 4.4. Immunofluorescence (Human Osteoblasts)

Human osteoblasts were grown on poly-L-lysine coated coverslips. At ~60% confluence, cells were transfected with the indicated siRNA as described below. Human osteoblasts were fixed with 4% (*w*/*v*) paraformaldehyde followed by 100% ice-cold methanol and were processed with the following antibodies: anti-CaSR (mouse monoclonal, Sigma clone HL1499), anti-Homer-1b/c (rabbit) and isotype controls. Coverslips were then washed and incubated with anti-rabbit Alexa Fluor 488 (1:750; Santa Cruz) and anti-mouse Cy3 (1:750; Life Technologies) at room temperature for 60 min. To visualize the nuclei, coverslips were mounted with UltraCruz™ Mounting Medium containing DAPI.

### 4.5. siRNA Transfection

siRNAs against all target transcripts (Homer1: sc-35581; CaSR: sc-44373 both from Santa Cruz Biotechnology) were used at a concentration of 50 nM in the presence of siRNA transfection reagent (Santa Cruz Biotechnology) according to the manufacturer’s instructions. Control transfections containing a non-directed siRNA sequence were carried out simultaneously, on the same plate, for all experiments. Briefly, osteoblasts were transfected overnight under serum free conditions, and permitted to grow for an additional 24 h in complete growth media after transfection, which allowed efficient reduction to the level of targeted protein. Protein knockdown was confirmed by Western blot. siRNA sequences are shown in Table S1 (Supplementary data). Individual siRNA duplex sequences used in isolation were not effective at achieving knockdown at the protein level in our primary human osteoblast cultures. For this reason, a pool of three siRNA sequences designed to target the same mRNA were used, and a significant level of protein knockdown was achieved that permitted functional analyses to be conducted on the cells. This finding was in agreement with previous comparisons of individual versus pooled siRNA duplexes [38].

### 4.6. Chemical Denaturation of Whole Mouse Bones Followed by Protein Level Measurement by Western Blot

Total protein was chemically extracted from mouse long bones as previously described [4].

### 4.7. qPCR mRNA Quantificiation

Homer1 and CaSR mRNA levels were quantified in cultured primary human osteoblasts as well as in cultured primary human osteoblasts where the expression of either Homer1 or CaSR were knocked down using siRNA transfection. Total RNA was extracted from cultured cells using the Isolate II RNA extraction kit (Bioline) and single stranded cDNA was synthesized using Superscript III reverse transcriptase (Invitrogen) according to manufacturer instructions. cDNA was subjected to real-time PCR using SensiFASTTM SYBR No-ROX kit (Bioline, London, UK) on a Rotor-Gene 6000 thermocycler (Corbett Research, Sydney, Australia) using cyclophilin D as a house keeping gene. Primers for the PCR reactions were: CaSR F: GAAAGACAGAATGTCACCAG; R: CATATGGTATAGGAGGGGTG, HOMER1: F:ACTGAAACTGAAGGAAGAGG; R: TTTCTGAGTCAAAGAATCCC, and cyclophilin D F: CAAATTGGCAGGGAGCAAT; R:ATCCTTGCCATCCTTGAGC. Relative expression changes of CaSR and Homer1 relative to cyclophilin D were calculated using the 2–∆∆Ct method. PCR reactions products were run on an agarose gel to confirm a single product of the correct size.

### 4.8. Western Blot Analyses

Equal amounts of total protein obtained from monolayer cultures of primary human osteoblasts, or chemically extracted from mouse long bones were analyzed by SDS-PAGE under reducing conditions followed by Western blot transfer to nitrocellulose membranes as previously described [3].

### 4.9. Statistics and Data Analysis

Experiments were performed in triplicate. Each experiment was repeated at least three times, with cells from different donors, and the data are reported as means ± standard deviation. One-way analysis of variance with the Tukey post-test were used to determine significant differences between treatments using Prism (GraphPad Software Inc., San Diego, CA, USA).

## Data Availability

Requests for data and materials collected from the study should be made to the corresponding author.

## Supplementary Materials

The following are available online at https://www.mdpi.com/article/10.3390/ijms22126509/s1.
