# Supplementary material for: The mTORC2 Regulator Homer1 Modulates Protein Levels and Sub-Cellular Localization of the CaSR in Osteoblast-Lineage Cells
Source: Int J Mol Sci. 2021 Jun 17;22(12):6509. doi: 10.3390/ijms22126509 (PMC8234890; doi:10.3390/ijms22126509)
Supplement: Supplementary file 1 [file ijms-22-06509-s001.zip › ijms-1230595-supplementary.pdf]

SUPPLEMENTAL TABLES

Table S1. siRNA duplex sequences obtained from Santa Cruz Biotechnology.

Gene	siRNA duplex manufacturer code	siRNA duplex sequence 5' to 3'
Homer1 (human)	A	Sense: UGAACUGGCUACCCUCAAAtt
		Antisense: UUUGAGGGUAGCCAGUUCAtt
	B	Sense: GCAACUUGCUGCCUAUCAAtt
		Antisense: UUGAUAGGCAGCAAGUUGCtt
	C	Sense: CCAAGCAAAUGCAGUACAUtt
		Antisense: AUGUACUGCAUUUGCUUGGtt
CaSR (human)	A	Sense: CUGGUUACAGGCUAUGAUAtt
		Antisense: UAUCAUAGCCUGUAACCAGtt
	B	Sense: CUUGCAACACCGUUUCUAAtt
		Antisense: UUAGAAACGGUGUUGCAAGtt
	C	Sense: GAAUGUAUCUCCUCCUAUUtt
		Antisense: AAUAGGAGGAGAUACAUUCtt

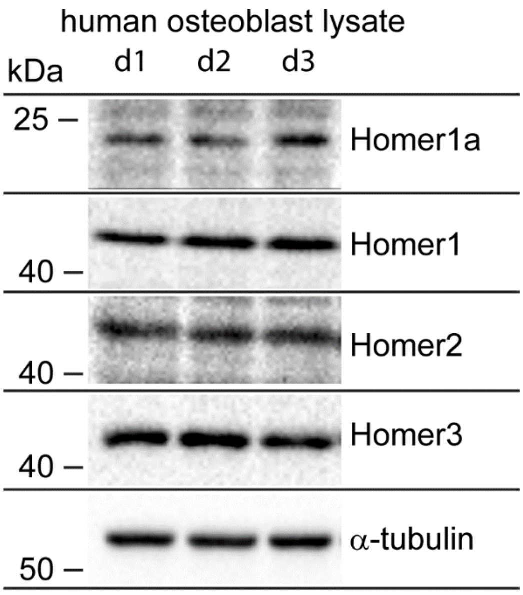

Figure S1. Multiple Homer isoforms are expressed in primary human osteoblasts. Primary human osteoblasts from three independent donors (d1-d3) were cultured in 10% DMEM for 48 h and lysed. Equal amounts of total cell protein were then analysed by western blot for Homer1a, Homer1, Homer2, Homer-3 or α-tubulin (loading control).

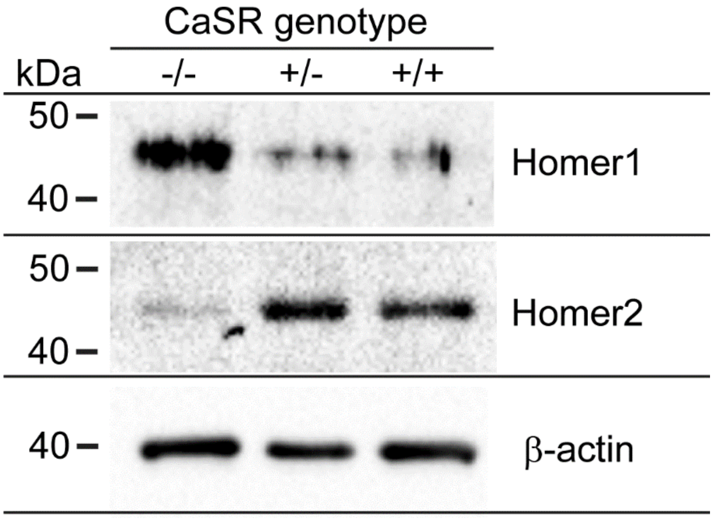

Figure S2. Decreased Homer2 protein levels were associated with overexpressed levels of Homer1 protein in the long bones of CaSR(-/-) mice. Western blot for Homer1, Homer2 or β-actin (loading control) of protein extracted from the long bones of wild type (+/+), heterozygous (+/-) or CaSR deficient (-/-) mice.

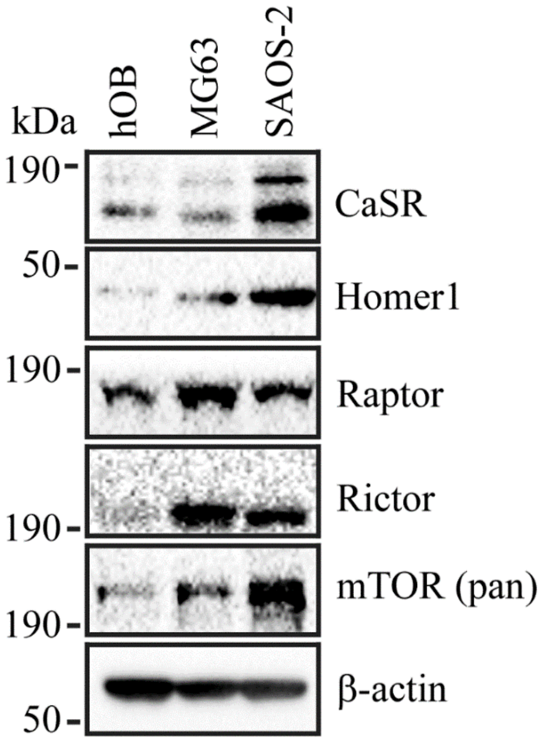

Figure S3. Human osteosarcoma cells overexpress Homer1 as well as CaSR and Rictor compared to primary human osteoblasts. Primary human osteoblasts (hOB), or osteosarcoma cells MG63 or SAOS-2 were cultured in 10% DMEM for 48 h and lysed. Equal amounts of total cell protein were then analysed by western blot for CaSR, Homer1, Raptor, Rictor, mTOR or β-actin (loading control).

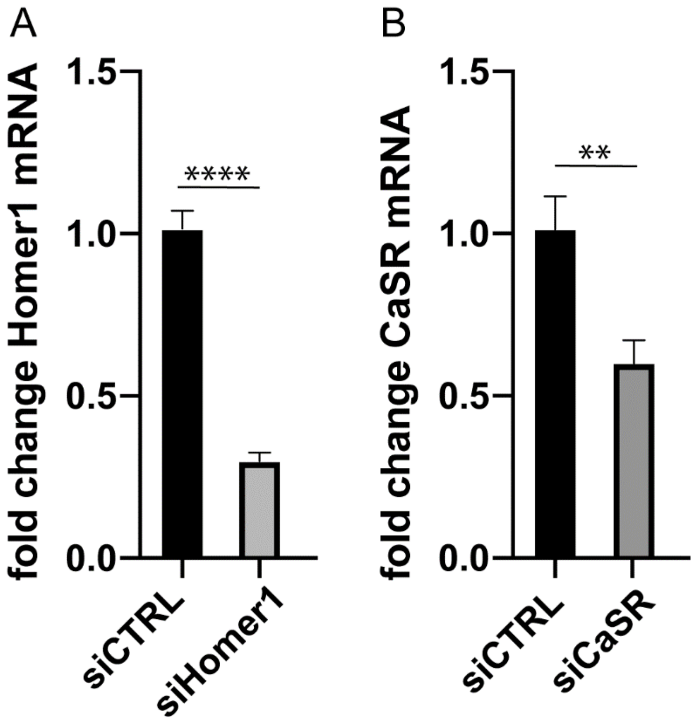

Figure S4. siRNA directed at Homer1 and CaSR resulted in significant reductions in target mRNA. Primary human osteoblasts grown in monolayers were transfected with siRNA (50 nM) directed at (A) Homer1 and (B) CaSR. 48 h post-transfection the relative mRNA level of each gene was determined by qPCR using the housekeeping gene cyclophilin D. Graphs are expressed as fold mRNA level of control transfected cells for each siRNA transfection.
